# Editorial comment on “Usefulness of peak frequency in electrograms for elimination of left atrial posterior wall residual potentials via epicardial connections”

**DOI:** 10.1002/joa3.13187

**Published:** 2024-11-21

**Authors:** Tetsuji Shinohara

**Affiliations:** ^1^ Department of Cardiology and Clinical Examination, Faculty of Medicine Oita University Yufu Japan

**Keywords:** atrial fibrillation, catheter ablation, epicardial connection, left atrial posterior wall, Omnipolar Technology Near‐Field algorithm

In this issue of the *Journal of Arrhythmia*, Ishikura et al.^1^ reported a case of persistent atrial fibrillation (AF) with residual endocardial conduction after left atrial posterior wall (LAPW) isolation. Patients with AF have been treated with either sinus rhythm maintenance (rhythm control) or adequate heart rate control (rate control) to improve symptoms. The EAST‐AFNET 4 trial^2^ showed that rhythm control is associated with better outcomes, at least in patients with early AF, and that rhythm control should be preferred in such patients. However, in catheter ablation of AF, pulmonary vein isolation (PVI) alone is not effective in maintaining sinus rhythm in some cases, especially in patients with persistent AF. Therefore, several strategies have been investigated in addition to PVI to reduce recurrent AF. Among them, the LAPW isolation has been widely performed with the promise of additional benefits. The LAPW isolation is performed by extending the PVI in lines along the roof and the bottom of the LAPW. However, in the KAPLA study by Kistler et al.,^3^ the addition of LAPW isolation to PVI in patients with persistent AF did not significantly improve freedom from atrial arrhythmias compared with PVI alone. On the other hand, the addition of LAPW isolation was reported to improve outcomes in patients with persistent AF who did not have low‐potential regions in the left atrium and in whom atrial arrhythmias were induced by continuous pacing.^4^ The exact reason for this discrepancy is unknown, but the re‐conduction on the LAPW isolation may be part of the cause. When LAPW isolation is performed, transmural conduction block by linear ablation of the left atrial roof and bottom remains challenging, mainly because of epicardial muscle fibers bridging epicardial and endocardial conduction, such as the septopulmonary bundle. Recently, the EnSite X mapping system became available with a new algorithm, the Omnipolar Technology (OT) Near‐Field algorithm (Abbott, St. Paul, MN). The algorithm can automatically annotate the highest peak frequency (PF) in local electrograms, resulting in accurate near‐field potential annotation.

In this issue, Ishikura et al.^1^ described that the residual potentials via epicardial connections (ECs) could be eliminated by using PF analysis of the OT Near‐Field algorithm. The conventional absolute *dV*/*dt* annotation algorithm annotates high‐amplitude electrograms. Therefore, the *dV*/*dt* algorithm probably may not accurately identify the location of an endocardial residual conduction gap in situations when both endocardial and epicardial electrograms are recorded together. In contrast, the OT Near‐Field algorithm using the PF value can identify whether the obtained electrograms are near‐ or far‐field signals. In fact, it has been reported that the OT Near‐Field algorithm can discriminate between far‐field and near‐field signals within complex electrograms.^5^ However, this algorithm has its drawbacks. If only far‐field potentials are recorded, it will annotate far‐field potentials. As a result, the appropriate ablation site cannot be identified. Ishikura et al. reported that the localization of the residual conduction gap in this case could be clearly visualized by increasing the cutoff frequency from 300 to 500 Hz. In the residual potentials of LAPW via ECs, their case study suggested that the higher PF electrograms may represent the area of conduction breakthrough from the epicardial to the endocardial layer. Therefore, when performing LAPW isolation, the OT Near‐Field algorithm is a useful tool that can distinguish between endocardial and epicardial signals. In their case, a PF threshold of 500 Hz was the optimal cutoff for locating the residual conduction gap. However, the optimal PF cutoff may differ depending on various factors such as patient characteristics and the direction of propagation of activation. Further studies are needed to determine the optimal cutoff for identifying the location of the conduction gap in LAPW isolation.

Again, although sinus rhythm is desirable in patients with AF, catheter ablation therapy with PV isolation alone is sometimes inadequate in patients with persistent AF. In such cases, additional ablation procedures including LAPW isolation are required. However, LAPW isolation is sometimes challenged by the presence of residual conduction gaps after LA linear ablation. The OT Near‐Field algorithm is a new algorithm that can visualize the localization of residual conduction gaps after LA linear ablation and is a useful tool for LAPW isolation.

## CONFLICT OF INTEREST STATEMENT

Author declares no conflict of interests for this article.
